# Der Stellenwert gesundheitsbewusster Ernährungsbildung in der Schule: Ergebnisse einer Analyse ausgewählter deutscher Rahmenlehrpläne

**DOI:** 10.1007/s00103-022-03544-9

**Published:** 2022-06-04

**Authors:** Louisa Prade, Matthias Richter, Gabriele I. Stangl, Uwe Hoßfeld, Astrid Fink, Anja Knöchelmann

**Affiliations:** 1 Institut für Medizinische Soziologie, Medizinische Fakultät, Martin-Luther-Universität Halle-Wittenberg, Magdeburger Str. 8, 06112 Halle (Saale), Deutschland; 2 Lehrstuhl für Social Determinants of Health, Fakultät für Sport- und Gesundheitswissenschaften, Technische Universität München, München, Deutschland; 3 Professur Humanernährung, Institut für Agrar- und Ernährungswissenschaften, Naturwissenschaftliche Fakultät III, Martin-Luther-Universität Halle-Wittenberg, Halle (Saale), Deutschland; 4 AG Biologiedidaktik, Friedrich-Schiller-Universität Jena, Jena, Deutschland; 5 Kreisverwaltung Groß-Gerau, Gesundheit und Verbraucherschutz, Groß-Gerau, Deutschland

**Keywords:** Kinder und Jugendliche, Übergewicht, Ernährungskompetenz, Ernährungsformen, Bildungspläne, Children and adolscents, Overweight, Nutrition literacy, Dietary forms, Education plans

## Abstract

**Einleitung:**

Übergewicht, auch bei Kindern und Jugendlichen, stellt national wie international ein großes Problem dar, welches stark mit Ernährung verknüpft ist. Ernährungsgewohnheiten werden bereits früh geprägt und können Einfluss auf die Krankheitsentstehung nehmen. Schulen als Lern- und Lebensorte können dabei ausgewogene und nachhaltige Ernährungsgewohnheiten fördern. Ziel der vorliegenden Arbeit war daher die Analyse ausgewählter Lehrpläne zur Ernährungsbildung (EB), um darauf basierend Defizite und Verbesserungsvorschläge für die schulische EB herauszuarbeiten.

**Methodik:**

Untersucht wurden naturwissenschaftliche und hauswirtschaftliche Rahmenlehrpläne weiterführender allgemeinbildender Schulen der neuen Bundesländer in der aktuellsten Version bis 2019 mittels qualitativer Lehrplananalyse. Im Fokus standen Ernährungsthemen sowie deren Vertiefungsmöglichkeiten, praktische Umsetzung und Zeitumfang.

**Ergebnisse und Diskussion:**

Ernährung und Humanbiologie werden vorrangig bis Klasse 8 in allen Lehrplänen unterrichtet, komplexere Themen größtenteils ab Klasse 9. Konkrete alltagsrelevante Lerninhalte zu praktischen Ernährungsweisen, zur Lebensmittelqualität sowie zur Rolle der Ernährung für die Krankheitsprävention können die Ernährungskompetenzen der Schüler:innen maßgeblich verbessern, finden jedoch derzeit zu wenig Beachtung in den Lehrplänen. Die Hauswirtschaftsfächer thematisieren u. a. Verbraucherthemen, werden aber nicht an Gymnasien bzw. nicht in allen Bundesländern angeboten. Wahlbereiche und Leistungskurse können die EB ergänzen, sind aber nicht an allen Schulen Teil des Lehrplans. Der Zeitumfang und die praktische Umsetzung für die EB sind u. a. stark von schulinternen Faktoren abhängig. Es lassen sich teilweise erhebliche Defizite in der EB feststellen.

**Schlussfolgerung:**

Als sinnvoll erachtet werden eine intensivierte EB in höheren Jahrgängen, die Einführung von Hauswirtschaftsfächern in allen Schulformen und eine Vereinheitlichung der Lehrplaninhalte zwischen den einzelnen Bundesländern.

## Abstract (en)

**Background:**

Obesity, including among children and adolescents, is a major problem both nationally and internationally and is strongly linked to nutrition. Eating habits are formed at an early age and can influence the development of disease. Schools as places of learning and living can promote balanced and sustainable eating habits. The aim of this study was therefore to analyze selected curricula on nutrition education in order to identify deficits and suggestions for improvement in school nutrition education.

**Methods:**

The most recent version of the scientific and home economics framework curricula of secondary schools as of 2019 were examined by means of qualitative curriculum analysis. The focus was on nutrition topics as well as their possibilities for in-depth study, practical implementation, and timeframe.

**Results and discussion:**

According to this analysis, nutrition and human biology are taught mainly up to grade 8 in all curricula, while more complex topics tend to be taught from grade 9 onward. Concrete learning content relevant to everyday life on practical nutrition, food quality, and the role of nutrition in disease prevention can significantly improve students’ nutritional literacy but currently receive too little attention in the curricula. Home economics subjects address consumer issues, among other topics, but are offered in only three out of six states. Electives and honors courses can supplement nutrition education, but are not part of the curriculum at all schools. The amount of time and the practical implementation of nutrition education are strongly dependent on internal school factors. In some cases, there are considerable deficits in nutrition education.

**Conclusion:**

It is considered useful to intensify nutrition education in higher grades, to introduce home economics subjects in all types of schools, and to standardize the curriculum content among the individual states.

## Einleitung

Gegenwärtig sind 15,4 % der Kinder und Jugendlichen in Deutschland übergewichtig, darunter 6,3 % adipös [1]. Dies ist vor allem vor dem Hintergrund problematisch, dass ein früh entwickeltes Übergewicht oft ein Leben lang bestehen bleibt und auch schon in jungen Jahren mit Zivilisations- und Folgeerkrankungen sowie psychosozialen Belastungen einhergehen kann [2, 3]. Zudem sind die Folgen sozial ungleich verteilt und es bestehen signifikante Zusammenhänge zwischen dem Auftreten von Übergewicht und assoziierten Erkrankungen abhängig von Bildungsstand und Schulform. Demnach gilt: Je höher der Schulabschluss, desto niedriger die Prävalenzen für Übergewicht und Adipositas sowie für daraus folgende Erkrankungen wie Bluthochdruck, koronare Herzerkrankung und Diabetes mellitus. Zudem sind die Prävalenzen je nach Wohnort unterschiedlich und gerade in den neuen Bundesländern auffällig hoch [1, 4–6].

Ein erster Schritt zur Vermeidung von assoziierten Erkrankungen stellt die Vermeidung von Übergewicht dar, wobei eine ausgewogene Ernährung neben körperlicher Aktivität eine zentrale Rolle einnimmt. Eine gesunde Ernährung ist für Heranwachsende außerdem zur Deckung von deren höherem Nährstoffbedarf relevant. Dabei stellt sich zunächst die Frage nach den Kenntnissen von Kindern und Jugendlichen über gesunde Ernährung. Internationale Studien zeigen, dass deren Wissen in diesem Bereich unzureichend ist, was ungesunde Ernährungs- und Lebensweisen begünstigt [7, 8]. Weitere Ergebnisse legen allerdings auch nahe, dass ein profundes Ernährungswissen nicht zwangsläufig mit einem gesünderen Ernährungsverhalten einhergeht [9, 10].

Kinder und Jugendliche verzehren einer deutschlandweiten Studie zufolge zu viel Wurst und Fleisch sowie Süßigkeiten, Limonaden und Snacks, während sie gleichzeitig die empfohlenen Konsummengen von Obst- und Gemüse nicht erreichen [11]. Aus einer Fehlernährung resultierende Folgekrankheiten und deren Behandlungskosten übersteigen in Deutschland mittlerweile 16,8 Mrd. € pro Jahr. Demnach stellen die Folgen falscher Ernährung nicht nur ein individuell medizinisches, sondern auch ein gesellschaftliches Problem dar [2, 12].

Daher sollte neben dem reinen Ernährungswissen die Ernährungskompetenz gezielt gefördert werden, welche zusätzlich zu den Kenntnissen auch ernährungsbezogene Fertigkeiten und Fähigkeiten einschließt [13]. Dies entspricht auch der Fokussierung auf den Kompetenzerwerb [12, 13], welcher durch die Formulierung der nationalen Bildungsstandards durch die Ständige Konferenz der Kultusminister der Länder (Kultusministerkonferenz, KMK) begonnen [13, 14] und in dem durch das Projekt REVIS (Reform der Ernährungs- und Verbraucherbildung in Schulen) entwickelten Referenzrahmen fortgesetzt wurde [15]. Ernährungsbildung (EB) ist demnach eines von 12 Handlungs- und Themenfeldern der Gesundheitsförderung [16] und stellt in diesem Zusammenhang einen wichtigen Ansatzpunkt dar, da sie die Lebensstilgewohnheiten stark beeinflusst [7, 17].

EB gehört zum Bildungsauftrag aller Bundesländer, wobei sich die praktische Umsetzung unterscheidet. Sie konzentriert sich auf verschiedene Möglichkeiten zur Verbesserung der Ernährungsgewohnheiten und umfasst neben dem Unterricht auch die Mahlzeitenverpflegung der Schüler:innen, Einflüsse von Lehrkräften und Eltern, Arbeitsmaterialien sowie schulische Rahmenbedingungen [18]. Studien konnten zeigen, dass Interventionsprogramme in der Schule signifikant zur Verbesserung von Ernährungswissen und -kompetenz beitragen können, v. a. wenn sie durch qualifizierte Fachkräfte, wie speziell ausgebildete Lehrkräfte oder zertifizierte Ernährungs- und Sportexpert:innen, durchgeführt werden und über einen längeren Zeitraum (≥ 6 Monate) erfolgen [19–25]. Für eine Verstetigung der EB können Rahmenlehrpläne (RLP) eine flächendeckende Basis darstellen.

Bereits 2004 stellten Heseker und Beer fest, dass für eine gute EB Folgendes benötigt wird: 1. Entwicklung eines neuen Kerncurriculums, 2. Einbettung der EB in die schulische Gesundheitsbildung, 3. Aus- und Fortbildung von Fachkräften sowie Erstellung qualitativ hochwertiger Materialien und Medien für die schulische EB [26]. Auch Jahre später ist dies nur teilweise erfolgt: EB ist zwar Bestandteil der Lehrpläne, nimmt jedoch keinen kontinuierlichen und umfassenden Anteil in der Schulbildung ein [27]. Dabei bleibt die Frage offen, welche konkreten Inhalte in den einzelnen Fächern und den dazugehörigen RLP enthalten sind.

Unser Ziel war daher eine detaillierte Analyse der inhaltlichen Ausrichtung zur EB in ausgewählten RLP weiterführender allgemeinbildender Schulen. Die neuen Bundesländer, inklusive Berlin, wurden hierbei fokussiert betrachtet aufgrund der besonders hohen Prävalenzen von Übergewicht und Adipositas in diesen Regionen. Die Schwerpunkte dieser Arbeit lagen dabei auf der inhaltlichen Ausgestaltung der schulischen EB und den Unterschieden zwischen den Schulen und Schulsystemen. Die Ergebnisse können als Basis für künftige Überarbeitungen der RLP dienen.

## Methodik

Im Rahmen des Projektes „Ernährung und Gesundheit im Schulfach Biologie“ des nutriCARD-Kompetenzclusters, welches vom Bundesministerium für Bildung und Forschung gefördert wird, wurden naturwissenschaftliche und hauswirtschaftliche RLP mittels qualitativer Lehrplananalyse systematisch auf ernährungs- und gesundheitsrelevante Themen und Inhalte untersucht. Die Lehrplananalyse wird als eine Form der Inhalts- und Dokumentenanalyse verstanden, bei der Lehrpläne mittels eines Kriterienkataloges auf Inhaltsangaben untersucht werden [28].

In die Analyse eingeschlossen wurden RLP allgemeinbildender weiterführender Schulformen der Sekundarstufen (Sek.) I und II der Bundesländer Berlin (BE), Brandenburg (BB), Mecklenburg-Vorpommern (MV), Sachsen (SN), Sachsen-Anhalt (ST) und Thüringen (TH). Im Fokus der Betrachtung standen RLP mit ernährungs-, gesundheits- oder hauswirtschaftsrelevanten Inhalten, darunter die naturwissenschaftlichen Fächer Biologie (Bio), Mensch-Natur-Technik (M-N-T), Naturwissenschaften (Nawi), Natur und Technik (N-T) sowie die Fächer Hauswirtschaft (HWS), Wirtschaft-Arbeit-Technik (WAT) und Wirtschaft-Technik-Haushalt/Soziales (WTH). Insgesamt ergaben sich daraus 21 RLP, jeweils in ihrer aktuellsten Version bis 2019.

Die RLP setzen sich jeweils aus verschiedenen Lernbereichen oder auch Themen zusammen, die entweder verpflichtend sind oder im Sinne eines Wahlbereiches oder Wahlthemas frei gewählt werden können. Letzteres hat Wahlpflichtcharakter. Darüber hinaus steht es den Lehrkräften frei, Schwerpunkte innerhalb eines Lernbereiches zu setzen. Da sich die Bezeichnungen in den untersuchten Bundesländern unterscheiden, dennoch aber eine einheitliche Beschreibung sinnvoll erschien, wurden die Begrifflichkeiten des Sächsischen Ministeriums für Kultus und Sport verwendet.

In der Voranalyse wurden ausgewählte RLP durch 3 Mitarbeiterinnen (AF, AK, LB) analysiert und relevante Inhalte extrahiert. Diese Ergebnisse wurden diskutiert und in Form eines Kategoriensystems zusammengefasst. Bei fehlender Übereinstimmung wurde eine weitere Person hinzugezogen. Dieses Kategoriensystem diente als Grundlage für die weitere detaillierte Extraktion gesundheits- und ernährungsbezogener Inhalte. Die dabei definierten Kategorien lauteten: (1) gesunde Ernährung, (2) gesunde Lebensweise und Nachhaltigkeit, (3) Lebensmittelproduktion von Anbau bis Konsum, (4) Lebensmittelqualität, (5) Ernährungsformen.

**Figure 1:**
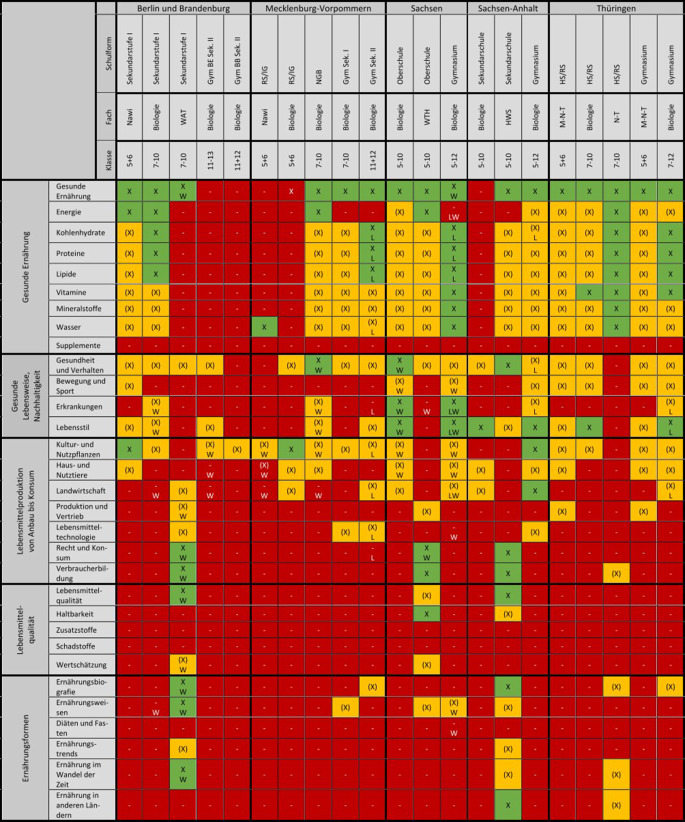

Jede der 5 Hauptkategorien beinhaltet mehrere definierte Unterkategorien, welche die inhaltlichen Aspekte darstellen (Abb. 1). Beispielsweise enthält die Kategorie (1) gesunde Ernährung u. a. die Unterkategorien „Fette“, „Vitamine“ und „Wasser“. Nach Erstellung der Kategorien und Unterkategorien erfolgte eine erneute Analyse der RLP, anhand derer die extrahierten Lehrplaninhalte in die entsprechenden Unterkategorien eingetragen wurden. Die visuelle Darstellung der Ergebnisse erfolgte anhand dreier Abstufungen in Tabellenform.

Konkrete und vollständige Lehrplaninhalte wurden mit einem „X“ gekennzeichnet (z. B. heißt es im Lehrplan: „Die Schüler:innen sollen sich Wissen zur Bedeutung von Vitaminen aneignen“, und die Vitamine werden direkt genannt). Indirekte und unvollständige Inhaltsangaben sind mit einem „(X)“ gekennzeichnet (z. B. „Die Schüler:innen sollen Kenntnisse über Nährstoffe und gesunde Ernährung gewinnen“, die Vitamine werden nicht direkt genannt). Nicht im RLP aufgeführte und somit offenbar nicht im Unterricht behandelte Lehrplaninhalte sind mit einem „−“ versehen. Fakultative Lehrplaninhalte und Wahlbereiche^1^ sind ergänzend mit einem „W“ gekennzeichnet, vertiefende Aspekte aus Leistungskursen mit einem „L“.

Separate Beachtung fanden die praktische Umsetzung (z. B. durch Experimente und Exkursionen), die Klassenstufen, in welchen die Lehrplaninhalte unterrichtet werden, sowie der vorgegebene Zeitumfang in Unterrichtsstunden.

## Ergebnisse

Die Ergebnisse der Untersuchung sind tabellarisch in Abb. 1 zusammengefasst.

### Kategorie (1) gesunde Ernährung.

Human- und verhaltensbiologische Themen wie Körper, Gesundheit und Ernährung werden unabhängig von Schulform und Bundesland vorrangig in den unteren Jahrgängen bis Klasse 8 unterrichtet. Zu Ernährung werden Schlagworte wie „Nährstoffe“ und „Lebensmittelpyramide“ angegeben, eine konkrete Definition einer ausgewogenen und vollwertigen Ernährung ist jedoch in keinem RLP vermerkt. Gleiches gilt für die in den RLP angesprochenen „Wirk- und Ergänzungsstoffe“. Der Fokus liegt bei den Nährstoffen hauptsächlich auf den Makronährstoffen, oft in Kombination mit experimentellen Nachweisreaktionen oder vertieft durch Wahlbereiche. Auch hier fehlen meist konkrete Lehrplaninhalte, z. B. zu Referenzwerten, Funktionen, Stoffwechselprozessen und Erkrankungen. Deutlich seltener wird auf die Aspekte Energiebedarf, Vitamine, Mineralstoffe und Wasser eingegangen. Nahrungsergänzungsmittel werden in keinem RLP genannt. Wie Abb. 1 zu entnehmen ist, enthalten die gymnasialen Oberstufen in BE/BB sowie der Biologielehrplan der Sekundarschule ST das Thema Ernährung nicht.

### Kategorie (2) gesunde Lebensweise und Nachhaltigkeit.

Bundeslandunabhängig enthalten fast alle RLP Angaben zur Reflexion des eigenen Gesundheitsverhaltens sowie zu gesunderhaltenden Maßnahmen. Auch hier fehlen konkrete Definitionen und Themenvorgaben. Die Unterkategorie „Bewegung und Sport“ wird zwar in den naturwissenschaftlichen RLP angesprochen, jedoch können nur in 8 der 21 RLP Bezüge zur empfohlenen Ernährung bei sportlicher Aktivität hergestellt werden.

Ebenso selten werden ernährungsbedingte Erkrankungen behandelt. Thematisch beschäftigt sich diese Unterkategorie lehrplanabhängig mit den Zusammenhängen zwischen Körperbau, Stoffwechsel und der Entstehung von Krankheiten wie Herz-Kreislauf-Erkrankungen, Diabetes mellitus, Adipositas, Allergien, Essstörungen und Krebs. Erkrankungen des Darms, der Leber, der Nieren sowie rheumatische Erkrankungen werden nicht behandelt. Wahlbereiche und Leistungskurse ergänzen den Themenbereich, v. a. in SN. Häufig genannt werden Auswirkungen von Lebensstilfaktoren und insbesondere der Suchtprävention (Tabak, Alkohol, Drogen), auf die Gesundheit des Nervensystems (SN, ST, TH).

### Kategorie (3) Lebensmittelproduktion von Anbau bis Konsum.

Entsprechend dem allgemeinen Lehrplanaufbau stehen ab Klasse 9 komplexe Themen wie Ökologie, Molekularbiologie und Genetik im Mittelpunkt des Unterrichts. Auch komplexere Aspekte der Lebensmittelproduktion werden in den höheren Jahrgängen behandelt (v. a. SN, ST). Die naturwissenschaftlichen Fächer beschäftigen sich u. a. mit Samenpflanzen, Tierwelt und Umweltfaktoren, selten wird jedoch konkret auf Nutzpflanzen, Nutztiere und die Landwirtschaft verwiesen. Ebenso finden sich nur wenige Angaben zu Produktionsverfahren und Lebensmitteltechnologie. Der Bereich Lebensmitteltechnologie ergänzt thematisch die Unterkategorie Lebensmittelqualität und geht v. a. auf den Einsatz von Mikroorganismen zur Konservierung von Lebensmitteln und bei Gärungsprozessen ein. Vereinzelt werden gentechnische Verfahren und Züchtung behandelt. Die Hauswirtschaftsfächer fokussieren sich auf die alltagsrelevanten Aspekte Verbraucher, Konsum, Haushalt und Küche. Wahlbereiche und Leistungskurse vertiefen Pflanzenbau, Tierhaltung, Formen der Landwirtschaft, Gentechnik und Konsum.

### Kategorie (4) Lebensmittelqualität.

Die Lebensmittelqualität wird nur rudimentär behandelt. Ausschließlich die Hauswirtschaftsfächer adressieren Themen wie Einkaufstraining, Produktkennzeichnung oder Qualitätsmerkmale von Lebensmitteln, allerdings nur bis Klasse 8.

### Kategorie (5) Ernährungsformen.

Der fünfte Themenbereich wird vorrangig von den Hauswirtschaftsfächern behandelt. Bundesländer, welche keine Hauswirtschaftsfächer aufweisen, greifen Ernährungsformen nur am Rande auf. Meist werden „Ernährungsweisen“ als Schlagwort genannt, aber auch hier fehlt eine konkrete Benennung, welche Ernährungsformen, Diäten und Trends im Unterricht behandelt werden. Einzelne Wahlbereiche (BE/BB, SN) beschäftigen sich ergänzend mit Vegetarismus und der Reflexion des eigenen Ernährungsverhaltens.

### Praktische Umsetzung.

Alle naturwissenschaftlichen RLP enthalten bundeslandunabhängig vereinzelt Angaben zu Unterrichtsformen, wie praktische Nachweise (z. B. Makronährstoffe, Gärung), Experimente (z. B. Enzymatik, Pflanzenwachstum) und Naturbeobachtungen (z. B. Pflanzenbestimmung, Mikroskop). Weniger als die Hälfte der RLP erwähnt Übungen zur Erfassung der Fitness, wie z. B. Vitalmessungen und Sportübungen, oder zur Lebensmittelsensorik (z. B. Verkostung, Qualitätsprüfung). Die Hauswirtschaftsfächer setzen den Fokus alltagsrelevant auf Planung, Lebensmitteleinkauf nach Qualitätsmerkmalen, Speisenzubereitung im Arbeitsbereich Küche, Beachtung der Küchenhygiene und Einnahme von gemeinsamen Mahlzeiten. Fakultativ werden in WAT Projekte vorgeschlagen, wie beispielsweise Kochwettbewerbe. Die RLP aus ST heben die Nutzung digitaler Medien und Technologien hervor (z. B. Ernährungs- und Gesundheits-Apps). Vereinzelt werden konkrete Unterrichtsformen vorgeschlagen, wie Exkursionen oder fächerverbindender Unterricht. Dagegen finden sich keine Angaben zu konkreten Unterrichtsformen wie Einzel- und Gruppenarbeiten, Präsentationen oder Planspielen.

### Zeitumfang.

BE/BB, MV (Sek. I), ST und TH geben in den RLP keine Zeitvorgaben für den Umfang der einzelnen Lern- und Wahlbereiche an. Sie weisen darauf hin, dass die Reihenfolge der Lernbereiche mehr oder weniger verbindlich und von verschiedenen Gegebenheiten abhängig ist. Im Gegensatz dazu enthalten die sächsischen RLP und der Oberstufenlehrplan Biologie MV Zeitvorgaben in Unterrichtsstunden, auch getrennt nach Grund- und Leistungskurs. Für den Lernbereich „Ernährung, Verdauung und Ausscheidung beim Menschen“ (RLP Biologie, Gymnasium SN) sind beispielsweise 10 Unterrichtsstunden vorgesehen [29].

## Diskussion

Aspekte einer ausgewogenen und gesundheitsfördernden Ernährung werden in allen Bundesländern und Schulformen in den RLP in unterschiedlichem Ausmaß und Tiefe aufgegriffen. Dabei werden Humanbiologie und Ernährung vorrangig bis Klasse 8 unterrichtet, komplexere Themen ab Klasse 9. Umfang und Komplexität der Themenfelder steigen dabei mit den Klassenstufen bzw. beim Vergleich von Mittelschule und Gymnasium. Während die naturwissenschaftlichen Fächer in Theorie und Praxis auf naturwissenschaftliche Aspekte abzielen, bieten die Hauswirtschaftsfächer eine multiperspektivische Betrachtungsweise. Sie behandeln theoretisch und praktisch die Bereiche Haushalt, Mahlzeitenzubereitung und Konsum. Obwohl viele Themenfelder bereits in den RLP verankert sind, gibt es auch Aspekte, die in den aktuell vorliegenden Versionen zu wenig Beachtung finden, um gezielt eine ausgewogene Ernährung und die Prävention von Übergewicht und seinen Folgeerkrankungen zu fördern.

Die Ergebnisse dieser Arbeit decken sich mit denen von Dankers et al., die zeigen konnten, dass EB zwar in allen Bundesländern Teil der RLP, aber nicht durchgängig und mehrperspektivisch in ihnen verankert ist [27].

Kenntnisse und Kompetenzen zu gesunder Ernährung besitzen besondere Relevanz, wenn es um ihre Anwendung im Alltag geht, wie dem Einkauf qualitativ hochwertiger Lebensmittel, der Zubereitung gesunder Mahlzeiten, der Prävention und Behandlung von Erkrankungen, dem Wechsel zu neuen Ernährungsformen und Diäten oder der Berücksichtigung von ökologischen Aspekten bei der Lebensmittelauswahl. Ein häufig vernachlässigtes Thema sind zudem Nahrungsergänzungsmittel, obgleich das Angebot von ihnen vielfältig ist. Da ihre Einnahme bei einer ausgewogenen Ernährung jedoch nicht notwendig ist, sollten sich die Schüler:innen mit Nutzen und Risiken solcher Präparate auseinandersetzen. Da schulinterne Curricula und Materialien variieren können, wäre es angeraten, konkrete Schlagwörter und Definitionen zum Themenfeld Ernährung in den einzelnen RLP zu ergänzen.

Ergänzung finden könnten auch Lerninhalte zu den Zusammenhängen von Ernährung, Stoffwechselprozessen und Krankheitsentstehung. Diese werden bislang hauptsächlich in den unteren Jahrgängen unterrichtet und folglich in Altersklassen, in denen die Mahlzeitenversorgung vorrangig durch Eltern oder Schulmensen erfolgt. Es ist daher fraglich, ob die Schüler:innen am Ende ihrer Schullaufbahn in Klasse 10 bzw. 12 auf umfassende alltagstaugliche Kenntnisse und Kompetenzen zu Ernährung zurückgreifen können, um später gesundheitsbewusst ihre eigene Versorgung zu gestalten.

Grundlagen der Verbraucherbildung und des Lebensmittelrechts werden ausschließlich in den Hauswirtschaftsfächern vermittelt. Da die Bundesländer MV und TH sowie Gymnasien keine Hauswirtschaftsfächer anbieten, besitzen sie an dieser Stelle Defizite in den Bereichen Verbraucherbildung, Konsum, Haushalt und Mahlzeitenzubereitung. Die Verbraucherbildung hat eine hohe Alltagsrelevanz, da sie die Menschen befähigt, Marketingstrategien zu durchschauen, Produktkennzeichnungen zu verstehen sowie Qualitätsmerkmale wie Haltbarkeitsangaben und Zugabe von Zusatzstoffen beurteilen und souverän Risikobewertung von Schadstoffen vornehmen zu können. Diese Inhalte sollten daher zwingend in die RLP aufgenommen werden.

Ebenso wie die Kategorie Lebensmittelqualität finden auch Ernährungsformen bislang kaum Beachtung. Veränderungen der Ernährungsweisen im Wandel der Zeit, ökologische und ethische Aspekte der Nahrungsmittelauswahl oder kulturelle Unterschiede werden wenig berücksichtigt. Zwar sprechen einige RLP „Ernährungsweisen“ an, charakterisieren diese jedoch nicht näher. Dabei gab es noch nie so viele Ernährungsformen, Diäten und Ernährungstrends wie heute. Auch Kinder und Jugendliche setzen sich immer häufiger mit diesen Themen im Alltag auseinander, z. B. durch soziale Medien oder den Einfluss von Mitschüler:innen aus anderen Kulturkreisen. Eine kritische Auseinandersetzung mit der Vielfalt der heutigen Ernährungsformen kann die Schüler:innen dazu befähigen, deren positive und negative Gesundheits- und Umweltfolgen abschätzen zu können sowie sich eigener Ernährungsgewohnheiten bewusst zu werden und diese zu hinterfragen.

Viele der oben angesprochenen Aspekte finden sich auch in dem Nationalen Aktionsplan Bildung für nachhaltige Entwicklung (NAP BNE) wieder [30]. Danach soll BNE langfristig und als Querschnittsaufgabe in den Bildungsstrukturen verankert und verantwortungsbewusstes Denken und Handeln ermöglicht werden. Schulen stellen hierbei zentrale Handlungsfelder dar. BNE sollte sowohl in der Aus‑, Fort- und Weiterbildung von Lehrkräften integriert als auch in den Lehr- und Bildungsplänen verankert werden. Dies wäre nicht nur im Interesse von Politik und Gesellschaft, sondern würde auch Schüler:innen für eine ernährungsphysiologisch wertvolle und bewusste Ernährung sensibilisieren, die zudem ein zunehmendes Engagement für eine nachhaltige Lebensweise zeigen.

In die schulische Bildung könnten z. B. die Nachhaltigkeitsziele „Gesundheit und Wohlergehen“, „nachhaltige/r Konsum und Produktion“ und „Maßnahmen zum Klimaschutz“ einbezogen werden. Mögliche Anknüpfungspunkte stellen dabei vorhandene Initiativen wie IN FORM, „Zu gut für die Tonne!“ oder „Gut Essen macht stark“ dar, auf die zurückgegriffen werden könnte. Erste Ansätze finden sich in den untersuchten RLP. Diese ließen sich jedoch deutlich ausweiten. Dabei besteht die Möglichkeit auf vorhandene Materialien und Unterrichtskonzepte (z. B. „Umwelt im Unterricht“) zurückzugreifen oder Multiplikator:innen einzubinden. Darüber hinaus könnten die schulischen Lehrkräfte als Multiplikator:innen fungieren.

Möglichkeiten zu einer entsprechenden Fort- und Weiterbildung bieten sowohl Angebote staatlicher wie auch privater Träger. Dies geschieht u. a. im Rahmen der oben genannten Initiativen, welche sich sowohl auf den konkreten Unterricht beziehen als auch inhaltliche Vorschläge umfassen, was von den Lehrkräften als besonders gewinnbringend erachtet wurde [31]. Eine mögliche Schwierigkeit zur flächendeckenden Einführung stellt dabei die Zuständigkeit der einzelnen Bundesländer für die Aus‑, Fort- und Weiterbildung der Lehrkräfte sowie für die Erstellung der RLP dar. Dies zeigt sich u. a. darin, dass die Verankerung von BNE im Sinne einer übergreifenden Strategie, der Implementierung in den RLP sowie der Lehramtsausbildung und der Angebote an Weiterbildungsprogrammen in den verschiedenen Bundesländern unterschiedlich stark vorangeschritten ist. Erfreulicherweise zeigt sich ein allgemeines Interesse der Kultusminister:innen dergestalt, dass sich die KMK als Mitgliedsinstitution der Nationalen Plattform Bildung für nachhaltige Entwicklung (NP BNE) an der (Weiter‑)Entwicklung von Fort- und Weiterbildungsmöglichkeiten beteiligt.

Wahlthemen, Wahlbereiche oder Leistungskurse stellen gute Möglichkeiten dar, die Lehrplaninhalte thematisch zu ergänzen und oben Genanntes zu vertiefen, finden sich jedoch nicht in allen RLP. So enthalten ST und TH keine Wahlmöglichkeiten und BE/BB nennen keine inhaltlichen Unterschiede für Grund- und Leistungskurse. Die untersuchten Vertiefungsmöglichkeiten erweitern v. a. die Kenntnisse zur Gesunderhaltung des Körpers, Erkrankungen, Lebensmittelproduktion in der Landwirtschaft und Ernährungsweisen. Da die in dieser Arbeit betrachteten RLP besonders bei diesen Themen Lücken aufweisen, wäre eine obligatorische Integration der Wahlthemen in die RLP empfehlenswert.

Obwohl in allen RLP auf Experimente, Exkursionen oder andere praktische Übungen hingewiesen wird, kann nicht abschließend beurteilt werden, ob und in welchem Umfang sie in den Unterricht einfließen. Mögliche Unterschiede ergeben sich u. a. durch interne Curricula, örtliche Bedingungen (z. B. Schulküchen, Schulgarten), finanzielle Mittel und Zeitmangel, die jedoch im Rahmen dieser Arbeit nicht erfasst wurden. Weiterhin sind die Hauswirtschaftsfächer zwar stark projekt- und praxisorientiert, allerdings könnte deutlicher gekennzeichnet werden, welche Lehrplaninhalte und Kompetenzen theoretisch (z. B. Angaben der Nährwertkennzeichnung) oder tatsächlich praktisch (z. B. Anwendung der Nährwertkennzeichnung beim Einkaufstraining) vermittelt werden sollen. Da Gymnasien keine Hauswirtschaftsfächer anbieten, könnte für sie erwogen werden, Mahlzeitenzubereitungen ebenfalls praktisch in den RLP und den Unterricht einzubeziehen, da die Zubereitung ausgewogener Mahlzeiten unabhängig von der Schulart alle Kinder und Jugendlichen betrifft. Hier kann ebenfalls auf vorhandene Angebote des NAP BNE zurückgegriffen werden, wie z. B. Exkursionen zu Demonstrationsbetrieben Ökologischer Landbau [30].

Zu diskutieren wäre letztlich, konkrete Zeitvorgaben für die einzelnen Lernbereiche in allen Bundesländern verpflichtend zu machen. Durch einen festgelegten Stundensatz könnte dem Thema Ernährung mehr Gewicht verliehen werden.

### Stärken und Schwächen

Die in dieser Studie durchgeführte detaillierte Inhaltsanalyse der RLP, welche nicht nur die Lehrplaninhalte beschreibt, sondern diese auch mit Klassenstufen, Praxis und Vertiefungsmöglichkeiten verknüpft, ermöglicht einen umfassenderen Überblick über die Ernährungsbildung in Schulen als die alleinige Betrachtung der Inhaltsvorgaben. Die Erkenntnisse zu den Schwächen der EB können Anhaltspunkte für die verbesserte und nachhaltigere Übergewichts- und Krankheitsprävention bei Kindern und Jugendlichen bieten.

Allerdings wurden in dieser Arbeit nur RLP untersucht. Diese geben zwar zu erwerbende Lehrplaninhalte und Kompetenzen verbindlich vor, stellen jedoch keine starre Checkliste dar, welche Lehrkräfte zu erfüllen haben. Um individuelle, schulische, finanzielle, örtliche und gesellschaftliche Gegebenheiten berücksichtigen zu können, entwerfen die Schulen eigene schulinterne Curricula. Folglich können zwischen den einzelnen Schulen eines Bundeslandes mehr oder weniger große Unterschiede bezüglich der Umsetzung und des Einsatzes von Unterrichtsmaterialien bestehen. Darüber hinaus sind Lehrpläne dynamischen Veränderungen unterworfen und die Ergebnisse dieser Arbeit stellen nur eine Momentaufnahme dar, da Lehrpläne je nach aktuellem Stand der Forschung, Anforderungen der Gesellschaft oder gesetzlichen Rahmenbedingung aktualisiert und verändert werden.

In dieser Arbeit wurden zudem nicht alle Bundesländer betrachtet. Aufgrund länderspezifischer Unterschiede sollten die Ergebnisse daher nicht für die gesamte Bundesrepublik verallgemeinert werden. Darüber hinaus untersucht die Arbeit nur eine Auswahl bestimmter Schulfächer. Es ist nicht auszuschließen, dass Fächer wie Chemie, Geografie oder Sprachen ebenso relevante Informationen zu Teilaspekten der Ernährung liefern. Diese Fächer sollten in weiterführenden Arbeiten in die Betrachtungen eingeschlossen werden. Gleiches gilt für einen Vergleich mit den RLP anderer Bundesländer.

Durch die alleinige Fokussierung auf die RLP konnte nicht geprüft werden, inwiefern diese tatsächlich mit dem Ernährungswissen und der Ernährungskompetenz der Schüler:innen korrelieren. Es ist nicht auszuschließen, dass durch den Spielraum, den die RLP bieten, Schulen und Lehrkräfte ernährungsbezogene Themen bereits deutlich detaillierter behandeln, als es auf den ersten Blick wirkt. Daher scheint auch eine weiterführende Untersuchung zur individuellen Ausgestaltung der Rahmenvorgaben sinnvoll. Diese konnte allerdings im Rahmen der vorliegenden Studie nicht durchgeführt werden. Um die tatsächliche Wissens- und Kompetenzvermittlung angemessen abbilden zu können, wäre eine weiterführende Untersuchung erforderlich.

## Schlussfolgerung und Ausblick

Gesunde Ernährung, Übergewicht und Krankheitsprävention sind so aktuell wie nie zuvor. Laut dem Ernährungsreport 2019 des Bundesministeriums für Ernährung und Landwirtschaft (BMEL) befürworten 95 % der Befragten, Ernährung als festen Unterrichtsbestandteil in der Schule zu integrieren [32]. Ende 2020 wurde eigens eine Petition dafür gestartet, Ernährung verpflichtend in die Lehrpläne aufzunehmen [33].

Aspekte einer gesundheitsbewussten Ernährung sind zwar in allen untersuchten RLP zu finden, dennoch werden nicht alle Schwerpunkte ausreichend behandelt. Insbesondere alltagsrelevante Themen mit dem Potenzial, das Ernährungsverhalten zu ändern, kommen hierbei zu kurz. Eine Einigung auf relevante Ernährungsthemen, konkrete Inhalte, Praxiselemente, Vertiefungsmöglichkeiten und Zeitvorgaben ist daher unerlässlich, um die schulische EB in Deutschland zu optimieren. Empfehlenswert wäre letztlich die Einführung von Hauswirtschafts-, ernährungs- oder verbraucherbildungsbezogenen Fächern in den Schulen und Bundesländern, die diese bislang nicht anbieten.

Zudem wäre eine Ergänzung um weitere Themen des NAP BNE wünschenswert, allerdings sollten sich die Unterschiede zwischen den einzelnen Bundesländern nicht weiter ausweiten. Das gilt sowohl für die Lehramtsausbildung als auch für die Weiter- und Fortbildung der Lehrkräfte sowie die Erarbeitung von Strategien zur langfristigen Einbindung von BNE in die Lehrpläne und das Angebot von Initiativen. Dies wäre vor allem vor dem Hintergrund, dass die KMK eine Mitgliedsinstitution der NP BNE ist, wünschenswert.

Bei der Implementierung sollte darauf geachtet werden, dass alle Bevölkerungsgruppen in gleichem Maße angesprochen werden. Wie eingangs erwähnt, unterscheiden sich die Übergewichts- und Adipositasprävalenzen nach Bildungsstand [1]. Daher ist es unerlässlich, dass in den Schulen ein Präventionsansatz gewählt wird, der den einzelnen Bevölkerungsgruppen gerecht wird und die bestehenden Bildungslücken entsprechend schließt.

Es ist zudem offen und sollte weiter untersucht werden, inwiefern die RLP tatsächlich zu einer verbesserten Ernährungskompetenz führen und welche Rolle die soziale Umgebung, wie Freunde und Familie, sowie der sozioökonomische Hintergrund spielen.

## Footnotes

1 Einige Lehrpläne bieten Wahl(pflicht)bereiche zusätzlich zu den Pflichtlernbereichen an. Je nach Interesse an dem Thema oder zur Verfügung stehender Unterrichtszeit können Wahl(pflicht)bereiche ausgewählt und in den Unterricht integriert werden. Wahl(pflicht)fächer wurden in dieser Arbeit nicht untersucht.
